# Parental Acceptability and Experiences of In-Bed Resuscitation at Birth: Findings From a Cross-Sectional Survey

**DOI:** 10.1016/j.jpedcp.2026.200222

**Published:** 2026-06-12

**Authors:** Maria Wilander, Li Thies-Lagergren, Anna Santesson, Katarina Patriksson, Heike Rabe, Ola Andersson, Katarina Ekelöf

**Affiliations:** 1 Department of Clinical Sciences, Pediatrics/Neonatology, Lund University, Lund, Sweden; 2 Department of Pediatrics, Hospital of Halland, Halmstad, Sweden; 3 Department of Health Sciences, Lund University, Lund, Sweden; 4 Department of Clinical Sciences, Child and Adolescent Psychiatry, Lund University, Sweden; 5 Department of Health Sciences, University West, Trollhättan, Sweden; 6 Department of Pediatrics, Brighton and Sussex Medical School, University of Brighton and University of Sussex, Brighton, UK; 7 Department of Neonatology, University Hospitals Sussex NHS Foundation Trust, Sussex County Hospital, Brighton, UK; 8 Department of Neonatology, Skåne University Hospital, Malmö/Lund, Sweden; 9 Department of Medicine, Karolinska Institute, Stockholm, Sweden

**Keywords:** acceptability, cross-sectional, intact cord, parents, resuscitation

## Abstract

**Objective:**

To assess parental acceptability and experiences of in-bed intact cord resuscitation (ICR) vs standard care during neonatal resuscitation in the Sustained Cord Circulation and Ventilation (SAVE) trial.

**Study design:**

A national cross-sectional electronic survey was administered 1 month postpartum (2022-2025). A questionnaire initially developed by Katheria et al was used to assess parental acceptability and experiences of ICR. The survey targeted parents of neonates enrolled in the SAVE trial, a randomized controlled trial investigating ICR for ≥180 seconds with standard care. Data were analyzed using descriptive and inferential statistics. Acceptability was compared between groups using χ^2^ tests, with ORs calculated to quantify effect size.

**Results:**

The response rate was ∼75% and the final analyses included 333 surveys. Most responses were received from mothers. Parents experiencing in-bed ICR reported greater opportunities to see, touch, and maintain proximity to their neonate (OR, 3.88; 95% CI, 2.32-6.48, for physical contact). The in-bed ICR group also expressed a stronger sense of safety and more positive perceptions of study participation compared with those whose neonates received standard care. No differences between the groups were observed in communication with healthcare professionals. Baseline maternal, perinatal, and neonatal characteristics were comparable between the groups.

**Conclusions:**

These findings demonstrate the psychosocial benefits of in-bed ICR, enhancing parental proximity, safety perceptions, and physical contact during neonatal resuscitation and stabilization. These results provide a vital parental perspective that complements clinical outcomes and supports future considerations of zero-separation practices for resuscitated neonates.

Implementing evidence-based methods, such as delayed cord clamping (CC) at birth, is influenced by various factors, including the characteristics of the intervention, organizational dynamics, the attitudes and knowledge of healthcare professionals, and patient or guardian perspectives.^1^, ^2^, ^3^ Important characteristics of the intervention include its relative advantage, ease of use, safety, and evidence base.^1^^,^^4^ Attention to the new parents' experiences of the birth of a nonvigorous neonate needing respiratory support is highly important to acknowledge. It has been described that parents’ greatest fear, when invasive procedures and resuscitation are needed, is being separated from their baby, leading to a compelling need to stay during resuscitation.^5^ In a qualitative interview study involving guardians of children of various ages, parents described the experience of forced separation during resuscitation. These parents emphasized their strong need to see their child in the moment of resuscitation.^6^ An integrative review of 9 studies on parental experiences of pediatric resuscitation identified that parents navigate conflicting emotions, require targeted communication and support, and find physical presence comforting during the resuscitation situation.^7^ In a study of premature neonates, being separated and hindered from seeing or touching their sick or premature neonate created a feeling of frustration, anger, and confusion over not being able to see the neonate earlier.^8^

It is estimated that ≤10% of neonates will need some kind of respiratory support at birth.^9^ Hospital policies and organizational arrangements in maternal and neonatal care often result in forced separation of neonates and their mothers.^10^^,^^11^ It has been argued that the need for resuscitation should no longer be accepted as a justification for separation at birth.^12^ For vigorous neonates, delayed CC is established and widely recommended.^13^ For compromised neonates, current guidelines recommend CC within 60 seconds of birth to allow the timely initiation of neonatal resuscitation. The guidelines state that intact cord resuscitation (ICR) may be performed when appropriate equipment and team experience is available, but emphasize the need for more evidence.^14^^,^^15^ Performing early CC deprives the neonate of placental transfusion, exacerbating an already vulnerable situation.^16^ An increasing body of research describes the possibility of performing ICR in nonvigorous neonates.^17^, ^18^, ^19^ The randomized controlled multicenter study Sustained cord circulation And Ventilation (SAVE) was initiated to develop and evaluate an in-bed ICR method.^20^

To understand the barriers to and facilitators of implementing ICR, it is crucial to obtain an accurate understanding of parents’ perceptions regarding the benefits and drawbacks of in-bed ICR for neonates requiring resuscitation.^21^ There is currently limited knowledge about parents' experiences when witnessing ICR of their nonvigorous newborn. Insights into parental experiences are essential for developing and implementing optimal resuscitation methods for neonates. This study aimed to assess parents’ acceptability and experiences of in-bed ICR vs standard care during neonatal resuscitation in the SAVE trial.

## Method

### Study Design

This cross-sectional substudy was conducted within the SAVE-study, a 2-arm hybrid type 1 randomized controlled trial (RCT) carried out in Sweden.^20^ As a part of this multicenter research program, data on parents’ acceptability and experiences were collected via a questionnaire from parents of neonates enrolled in the primary randomized trial.

### The SAVE Study

#### Setting and Participation

The primary study was conducted across 9 neonatal units in Sweden between 2019 and 2024, representing all levels of the Swedish Neonatal Quality Register (level I, basic care without a dedicated neonatal ward; level II, county hospital providing special neonatal care from 28 gestational weeks; and level III, regional center with full neonatal intensive care). This substudy uses data from parents of children enrolled between March 2022 and December 2025. All study sites were publicly funded facilities, standard for Swedish births. Care was provided by multidisciplinary teams comprising obstetrical and neonatal healthcare professionals. Women with vaginal, singleton pregnancies at ≥35^0/7^ gestational weeks were enrolled. Neonates requiring resuscitation at birth formed the study cohort; their parents served as respondents in this survey study. Exclusion criteria were major malformations, compromised cord circulation, or emergency cesarean section.

#### Randomization and Blinding

Neonates were randomized 1:1 using sealed opaque envelopes, opened shortly before birth, to receive care according to the SAVE method (intervention group) or resuscitation initiated after CC, within 60 seconds after birth (control group). Blinding was not possible due to the nature of the intervention. Researchers were aware of group allocation (intervention vs control) when analyzing implementation (parent attitudes) outcomes.

#### The Intervention (SAVE Method)

The SAVE method is an approach to neonatal resuscitation performed in the mother’s bed with an intact umbilical cord for ≥3 minutes after birth, using standard resuscitation equipment. If resuscitation was needed beyond 3 minutes, or if more advanced interventions were required, the umbilical cord could be clamped and the neonate transferred either to a resuscitation table in the birthing room or to an adjacent emergency room. Alternatively, at the discretion of the attending physician, resuscitation could proceed with the cord remaining intact.

#### Standard Care (Control Group)

Standard care consisted of neonatal resuscitation measures performed after CC within 60 seconds after birth. Resuscitation was typically carried out on a dedicated resuscitation table, either in the labor room or in an adjacent area, where the other parent typically accompanies the neonate. The setting depended on local routines and space availability.

### Survey Study

#### Participants and Recruitment

Parents of neonates requiring resuscitation in the multicenter SAVE RCT were eligible. The contact email address provided at the time of informed consent was used to send a single electronic survey link 1 month after birth via the EnterMedic research platform. Participants self-identified as mother or father/partner within the survey. Responses from either parent were included. Maternal characteristics were collected from The Swedish Pregnancy Register.^22^ Information on partner characteristics was not available.

#### Outcome Measures

We used an adapted version of Katheria et al's instrument to assess parental acceptability of bedside resuscitation with an intact cord.^23^ The instrument is composed of 10 questions regarding participating in the research study, their overall satisfaction with the resuscitation situation, their ability to see or touch their neonate, and communication with healthcare professionals. Items 4-10 were rated on a Likert scale ranging from 0 to 4 depending on the question. The instrument was translated from English to Swedish.^24^ A multidisciplinary implementation group consisting of professionals from the vaginal birth setting was engaged in a Delphi process to adapt the instrument to the setting as further described in the study protocol.^20^ The multidisciplinary implementation group comprised members of the neonatal team (pediatrician, neonatologist, neonatal nurse), the labor team (midwife, obstetrician, head of department representative), and an implementation researcher. Items 4a and 4b were adapted for the Swedish context while preserving the integrity of the original item; the word “comfortable” was replaced with “safe and secure.” All other items were simple translations of the original. A stepwise forward-backward translation approach was used.

### Statistical Analyses

Analysis was performed using SPSS Statistics for Windows, Version 28.0 (IBM Corp). Before the analyses, we examined background variables and survey items for missing values, outliers, and normality of distribution (Supplementary Table S1; available at www.jpeds.com). Maternal and neonatal characteristics were cross-checked against the Swedish Pregnancy Register.^22^ Randomized group assignment was compared with the recorded CC time in the SAVE database to identify protocol violations and to determine actual exposure (in-bed treatment vs separation within the first minute).

Participants were grouped as “as-treated” based on actual CC times recorded in the SAVE RCT database. In this context, CC duration was used as a proxy for the actual exposure to bed-side resuscitation. Groups were defined as in-bed ICR (CC at ≥180 seconds), uncertain place of resuscitation (CC at 61-179 seconds), and standard care (CC at ≤60 seconds). Additional analyses were performed according to intention-to-treat (original RCT) and parent-reported place of resuscitation (Supplementary Tables S2 and S3; available at www.jpeds.com). Descriptive analyses summarized participant characteristics and questionnaire responses across all 3 groups. Data were presented as numbers (percentages), means (SD), or medians (Q1-Q3) as appropriate based on distribution. Demographic variables were recoded and items 4-10 were dichotomized (Likert scale of 0-2 vs 3-4) for comparative analysis. Comparisons of proportions were performed using Pearson’s χ^2^ test. Effect sizes were calculated using ORs with 95% CIs, values of 1.5, 2.4, and 4.0 correspond roughly with small, moderate, and large effects, respectively. Statistical significance was defined as a *P* value of <.05. Reporting of results was guided by the Strengthening the Reporting of Observational Studies in Epidemiology statements.^25^^,^^26^

### Ethical Considerations

The survey study was approved by the Swedish Ethical Review Board (Dnr. 2019-02368 with amendment 2021-06284-02). Written, informed consent from both parents was required for participation in the SAVE study. The parents' participation in the survey study was voluntary and could be terminated by them at any time, without any explanation.

## Results

Between April 2022 and January 2025, parents from all 9 study sites completed and returned a total of 345 questionnaires, for a response rate of 74.7%. The neonates included in the study were born between January 2022 and November 2024. Participant flow to final analysis (n = 333) is shown in the Figure.

**Figure:**
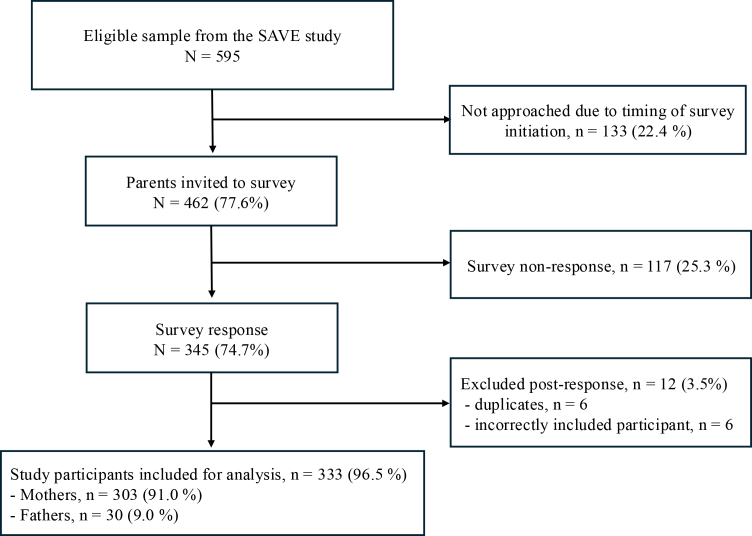
Participant flow from eligible to final analysis.

### Study Population and Response Overview

The majority of questionnaires (91%) were completed by mothers. Maternal and perinatal characteristics were generally comparable between groups (Table I). Almost 70% of the included mothers were aged between 25 and 35 years, were most likely to have a body mass index of <30, were born in Sweden, had completed college or university education, and were primiparous. Among the included mothers, almost 47% had their labor induced and about 67% experienced noninstrumental birth. Less than 8% of included mothers rated their birth experience below 4 on a 10-point visual analogue scale. The included neonates were mostly born between gestational weeks 37^0/7^ and 41^0/7^, in cephalic presentation, were male, and of a normal birth weight. Neonatal characteristics showed minor baseline differences between groups (gestational age >41 weeks and use of positive-pressure ventilation). All other maternal and neonatal variables were comparable.

**Table I:** Maternal, perinatal, and neonatal characteristics, categorized by actual exposure to site of resuscitation

Characteristics	In-bed ICR (CC ≥ 180s; n = 186 [55.9%])	Uncertain place of resuscitation (CC > 60s < 180s; n = 33 [9.9%])	Standard care (CC ≤ 60s; n = 114 [34.2%])
Maternal characteristics (n = 303)			
Age, years (n = 302)			
<25	6 (3.6)	0 (0)	4 (3.8)
25-35	108 (65.1)	19 (61.3)	78 (74.3)
>35	52 (31.3)	12 (38.7)	23 (21.9)
Body mass index (n = 291)			
<30	127 (79.4)	27 (87.1)	75 (75.0)
≥30	33 (20.6)	4 (12.9)	25 (25.0)
Country of birth (n = 296)			
Sweden	134 (91.2)	28 (93.3)	82 (87.2)
Other country	13 (8.8)	2 (6.7)	12 (12.8)
Level of education (n = 303)			
Elementary/high school	50 (30.1)	8 (25.0)	30 (28.6)
College/university	88 (53.0)	21 (65.6)	56 (53.3)
Not known	28 (16.9)	3 (9.4)	19 (18.1)
Parity			
Primipara	105 (64.0)	17 (54.8)	62 (60.8)
Multipara	59 (36.0)	14 (45.2)	40 (39.2)
Perinatal characteristics (n = 333)			
Onset of birth (n = 327)			
Spontaneous	106 (57.6)	14 (43.8)	55 (49.5)
Induction	78 (41.9)	18 (56.3)	56 (50.5)
Birth mode (n = 307)			
Noninstrumental birth	121 (68.7)	22 (71.0)	62 (62.0)
Instrumental delivery	55 (31.3)	9 (29.0)	38 (38.0)
Overall birth experience (n = 233)			
Visual analogue scale > 4	125 (92.6)	19 (95.0)	71 (91.0)
Neonatal characteristics (n = 333)			
Gestational age at birth, weeks (n = 327)			
<37	10 (5.4)	3 (9.4)	7 (6.3)
37-41	158 (85.9)	23 (71.9)	79 (71.2)
>41	16 (8.7)	6 (18.8)	25 (22.5)
Birth weight, g (n = 323)			
<2500	2 (1.1)	0 (0)	3 (2.7)
2500-4500	167 (92.8)	30 (93.8)	94 (84.7)
>4500	11 (6.1)	2 (6.3)	14 (12.6)
Sex			
Male	116 (63.0)	16 (50.0)	65 (58.6)
Apgar score			
1 min	6 (5-7)	7 (6-7)	5 (4-6)
5 min	8 (7-9)	8 (7-9)	8 (7-9)
Neonatal resuscitation interventions, n = 333			
Continuous positive airway pressure	154 (82.8)	22 (66.7)	85 (74.6)
Positive-pressure ventilation	77 (41.4)	11 (33.3)	58 (50.9)
Extra oxygen	103 (55.4)	14 (42.4)	51 (44.7)

Data are presented as number (%) or median [IQR]. Groups are defined by actual exposure using CC time as a proxy for resuscitation site.

Approximately 45% of participants reported being informed about the original study at antenatal clinics, and 42% first became aware of the study upon arrival at the labor ward. Among included respondents, 186 (55.9%) had neonates who received in-bed ICR, 114 (34.2%) had neonates who received standard care, and for 33 (9.9%) cases, place of resuscitation was uncertain.

### Parental Acceptance and Experiences With Clinical Environment and Care

Descriptive statistics and response distributions for each item are presented in Table II and Supplementary Table S1. Parents reported experiences with clinical care aspects such as visual and physical contact, communication with healthcare professionals, and perceived safety during resuscitation measures. In the total cohort (n = 333), median responses were 3 across nearly all items, although the IQRs varied. Out of all the items physical contact had the lowest median of 2 (0-4) among all responders, but with significant differences between standard care and in-bed ICR: median, 1 (Q1-Q3, 0-3) and median, 3 (Q1-Q3, 1-4), respectively (*P* < .001). The greatest proportion of strongly negative responses (41.2%) was seen for physical contact in the control group, and the greatest proportion of strongly positive responses (46.2%) was seen for visual contact in the in-bed ICR group.

**Table II:** Response distribution across response categories by item

Response alternatives	0	1	2	3	4	Median (Q1-Q3)
4 (a) If your child received support/resuscitation *in the delivery bed* or on the resuscitation table close to the mother how safe and secure were you with it?	1 (0.7)	4 (2.7)	27 (18.1)	50 (33.6)	67 (45.0)	3 (3-4)
4 (b) If your child received support/resuscitation *on the resuscitation table* in another room, how safe and secure were you with it?	3 (1.7)	14 (8.0)	57 (32.4)	69 (39.2)	33 (18.8)	3 (2-3)
5. Which of the following best describes your feeling about participating in the SAVE trial?	25 (7.5)	13 (3.9)	47 (14.1)	98 (29.4)	145 (43.5)	3 (2-4)
6. How do you think your participation in the SAVE trial has affected your child's health?	5 (1.5)	9 (2.7)	110 (33.0)	124 (37.2)	85 (25.5)	3 (2-4)
7. How much visual contact did you have with your baby immediately after birth?	41 (12.3)	58 (17.4)	40 (12.0)	73 (21.9)	121 (36.3)	3 (1-4)
8. How much were you able to touch your baby immediately after birth?	96 (28.8)	48 (14.4)	41 (12.3)	51 (15.3)	97 (29.1)	2 (0-4)
9. How well did the neonatal team communicate with you during the first few minutes when your child received support interventions?	10 (3.0)	38 (11.4)	64 (19.2)	95 (28.5)	126 (37.8)	3 (2-4)
10. Based on your experience with the SAVE trial, how likely are you to consider participating in another research study in the future?	11 (3.3)	4 (1.2)	30 (9.0)	115 (34.5)	173 (52.0)	4 (3-4)

Distribution of responses across categories (0-4) for each individual Likert scale item, presented in questionnaire order. Data are shown for total sample (n = 333).

Missingness was 0% for all items except item 5 (1.5%). For item 4, response options ranged from not safe and secure to completely safe. For items 5 and 6, response options ranged from strongly negative to strongly positive. For items 7 and 8, response options ranged from not at all to to a large extent. For item 9, response options ranged from very poor to excellent. For item 10, response options ranged from very unlikely to very likely. Medians with 25th percentile (Q1) and 75th percentile (Q3) are presented.

Table III presents comparisons between groups. A greater proportion of parents in the study reported feeling safe and secure when resuscitation was performed in bed (74.7%), compared with standard care (56.1%). The difference was of moderate magnitude (OR, 2.31). A greater proportion of parents in the in-bed ICR group also reported greater opportunities for visual and physical contact, with moderate effect for visual contact (OR, 2.62) and a large effect for physical contact (OR, 3.88). No significant differences were found between groups in parents’ perceptions of communication with healthcare professionals during resuscitation.

**Table III:** Dichotomized comparative analyses by actual clinical experience (as-treated)

Variables	In-bed ICR (CC ≥ 180s; n = 186)	Standard care (CC ≤ 60s n = 114)	OR (95% CI)	*P* value
Parental acceptance of the intervention				
How safe and secure did you feel about your baby receiving support in the warming/delivery bed?				
“Very safe and secure” to “Completely safe”	136 (74.7)	64 (56.1)	2.31 (1.40-3.80)	<.001
“Not safe and secure” to “moderately safe”	46 (25.3)	50 (43.9)		
How much visual contact did you have with your baby immediately after birth?				
“Considerably” to “To a large extent”	125 (67.2)	50 (43.9)	2.62 (1.62-4.24)	<.001
“Not at all” to “To some extent”	61 (32.8)	64 (56.1)		
How much were you able to touch your baby immediately after birth?				
“Considerably” to “To a large extent”	106 (57.0)	29 (25.4)	3.88 (2.32-6.48)	<.001
“Not at all” to “To some extent”	80 (43.0)	85 (74.6)		
How did you experience the staff communication with you during the baby’s first support minutes?				
“Good” to “Excellent”	130 (69.9)	70 (61.4)	1.46 (0.89-2.38)	.13
“Very poor” to “Fair”	56 (30.1)	44 (38.6)		
Parental acceptance of research∗				
How would you describe your feeling about participating in the SAVE trial?				
“Positive” to “Strongly positive”	152 (81.7)	69 (60.5)	3.14 (1.83-5.39)	<.001
“Neutral”	21 (11.3)	24 (21.1)		
“Strongly negative” to “Negative”	13 (7.0)	21 (18.4)		
What impact do you feel participating in the SAVE trial had on your baby’s health?				
“Positive” to “Strongly positive”	140 (75.3)	58 (50.9)	2.94 (1.79-4.82)	<.001
“Neutral”	40 (21.5)	51 (44.7)		
“Strongly negative” to “Negative”	6 (3.2)	5 (4.3)		
Given your experience of the SAVE trial, would you consider participate in another research study in the future?				
“Somewhat likely” to “Very likely”	162 (87.1)	96 (84.2)	1.27 (0.65-2.45)	.49
“Neutral”	15 (8.1)	13 (11.4)		
“Very unlikely” to “Somewhat unlikely”	9 (4.8)	5 (4.4)		

ORs (95% CI) for dichotomized outcomes (Likert response 0-2 vs 3-4), by “as-treated” group defined by CC time (in-bed ICR ≥ 180 s vs standard care ≤ 60 s). Responders with uncertain place of resuscitation were excluded from these analyses.

Effect size: <1.5 = small effect size, 2.4 = moderate effect size, >4.0 = large effect size.

∗ Neutral responses within these questions were dichotomized together with negative responses for comparing analyses.

### Parental Reflections and Acceptability of Study Participation

Most participants, 243 (74.1%) rated their participation in the SAVE RCT as positive or strongly positive, and 288 (86.5%) stated that they would be likely to participate in another study in the future based on their experience from the SAVE trial. For research items 5, 6, and 10 in the total cohort, responses were positive with medians of 3-4 (Table II). The greatest proportion of strongly positive responses (55.4%) among items of research was seen in the in-bed ICR group, for willingness to participate in future studies (Table II and Supplementary Table S1).

Parents who experienced in-bed ICR were significantly more likely to report positive feelings about participating in the SAVE trial and believed to a greater degree that the SAVE trial had a positive impact on their child’s health. The effect size differences were moderate for both items. No significant difference was found between the groups regarding attitude toward participation in future studies. The primary comparative analyses and effect sizes for all items are presented in Table III. The additional analyses performed according to intention-to-treat (original RCT) and parent-reported group affiliation confirmed similar effect sizes and significance levels to primary analyses (Supplementary Tables S2 and S3).

## Discussion

This is the first large-scale study to describe parents’ perceptions of witnessing neonatal resuscitation at birth within a multicenter RCT. Parents who experienced in-bed ICR reported an overall more positive experience, such as greater opportunities to see and touch their newborn neonate and a stronger sense of safety, compared with those whose neonates received standard care. These parents also expressed a more positive perception of their participation in the study. No differences were observed regarding communication with healthcare professionals.

The greater opportunities to see and touch their newborn strongly supports zero-separation and family-centered care principles, aligning with Cochrane evidence on skin-to-skin contact benefits for bonding and stability, as well as Sawyer et al’s findings that parents value proximity during resuscitation.^27^^,^^28^ The large effect size observed for parental ability to touch their infant underscores the profound clinical significance of this outcome. Although the timing of formal skin-to-skin contact was not recorded, the enhanced proximity provided by in-bed ICR likely facilitates a swifter transition to such contact post stabilization. Clinically, these differences highlight how in-bed ICR more effectively facilitates parental inclusion and physical closeness during neonatal resuscitation compared with standard care. However, successful adoption requires targeted, continuous training among healthcare professionals to build confidence in bedside procedures including in-bed ICR across different settings.^29^^,^^30^

Parents in our study also reported significantly higher feelings of safety and security during in-bed ICR compared with standard care. This finding is consistent with a UK usability study reporting positive parental feedback on closeness and witnessing airway management during trolley-based ICR.^31^ Katheria et al reported results of similar outcomes in a pilot RCT investigating the acceptability of using a resuscitation platform to perform ICR in 60 term neonates.^23^ However, our findings differ from those of Katheria et al, where fewer than one-half of parents reported feeling safe during neonatal resuscitation. This discrepancy may reflect differences between in-bed ICR and bedside trolley ICR, but neonatal characteristics and intervention levels may contribute. Although most parents in our study accepted in-bed ICR, more than one-fifth in the in-bed ICR group still reported not feeling very safe, highlighting persistent anxiety related to neonatal resuscitation, regardless of method. This finding aligns with Sawyer et al’s English interview study regarding a resuscitation platform, where parents valued ICR for reassurance, visibility of care, and family involvement, yet some reported negative emotions from witnessing interventions.^28^ Persistent parental anxiety during neonatal resuscitation may stem from the distress of a neonate needing life-saving support or from the intimate in-bed ICR setting, which some professionals perceive as potentially vulnerability inducing for mothers.^30^ In contrast, parents themselves appreciated professionals’ focused care.^3^ These perspectives highlight in-bed ICR’s role in promoting family participation and zero separation, as in midwives’ practices.^29^

Parents are vital stakeholders in developing novel methods involving neonatal care and including neonates in research, which is essential to ensure the development of safe, evidence-based care and treatments.^32^ Rodriguez et al noted nearly 2 decades ago that parental perspectives on pediatric research participation were largely overlooked, underscoring the need to incorporate these viewpoints into recruitment strategies.^33^ In our study, parents valued SAVE trial participation, irrespective of allocation, with most responders reporting perceived positive effects on their neonate’s health, particularly among parents in the in-bed ICR group. Parents have historically been assumed to be concerned about trial involvement, especially when there are perceived group advantages.^33^ Our findings challenge this assumption, demonstrating positive parental perceptions of research participation that may help researchers to generate outcomes relevant to parents and potentially enhance future neonatal trial recruitment. Consistent with this result, a qualitative study with focus groups showed that patient and public involvement in neonatal research fosters mutual learning between parents and researchers.^34^

### Strengths and Limitations

The present study was nested within the SAVE RCT, enabling a controlled setting evaluating both clinical and implementation outcomes.^20^ Although the survey is based on the SAVE RCT, the responses are parental responses and not objective measures. However, there are no objective data available to assess the parental experiences in the resuscitation situation. A major strength is the large cohort size (n = 333), representing the largest systematic assessment to date of parental experiences with in-bed neonatal ICR. The SAVE trial was carried out across 9 Swedish hospitals with varying neonatal intensive care unit levels, enhancing generalizability. Findings remained consistent across the 3 different analytical approaches: primary as-treated based on CC time as a proxy for location, original randomized allocation (intention to treat), and parent-reported place of resuscitation. Despite numerical imbalances in the as-treated group analyses due to protocol violations in the original study, the consistency of results across different group classifications supports the robustness of our findings and suggests that this imbalance did not significantly bias the outcomes. The high response rate (∼75%) among approached parents minimizes nonresponse bias and ensures representativeness. In addition, the use of a previously applied instrument allows comparison with earlier CC studies.

Several limitations should be acknowledged. Most neonates received only brief respiratory support (continuous positive airway pressure or positive-pressure ventilation), limiting generalizability to advanced resuscitation scenarios such as chest compressions. Parents volunteered for the SAVE RCT, potentially introducing volunteer bias. They could not be blinded to the intervention due to its nature, which may also have influenced survey responses. This potential selection bias, as well as the risk of recall bias from retrospective reporting awareness, further warrant caution in interpretation. We chose to include responses from either parent to capture a broader family-centered perspective. Although differences between maternal and partner experience are likely, a marked imbalance in responses precluded meaningful comparisons. Furthermore, we cannot exclude the possibility that some responses reflect a combined parental perspective. Parents’ representatives were not involved in questionnaire development, despite calls for codesigned patient-reported outcome measures to capture important issues from patients’ perspectives.^35^ Future studies should involve parental representatives who have personal experience of neonatal resuscitation interventions.

## Conclusion

Parents experiencing in-bed neonatal resuscitation with an intact umbilical cord reported significantly more positive experiences and study participation perceptions compared with standard care. Our findings support zero-separation practices for neonates requiring resuscitation, a population often excluded from family-integrated models, by demonstrating the psychosocial benefits of in-bed ICR in maintaining parental proximity. Further research should explore these effects in neonates requiring more advanced resuscitation interventions.

## Declaration of Generative AI and AI-Assisted Technologies in the Manuscript Preparation Process

During the preparation of this work the authors used Perplexity AI, San Francisco, CA, for language editing and phrasing suggestions. After using this tool/service, the authors reviewed and edited the content as needed and take full responsibility for the content of the published article.

## CRediT authorship contribution statement

**Maria Wilander:** Writing – review & editing, Writing – original draft, Methodology, Funding acquisition, Formal analysis, Data curation, Conceptualization. **Li Thies-Lagergren:** Writing – review & editing, Writing – original draft, Supervision, Methodology, Formal analysis, Data curation, Conceptualization. **Anna Santesson:** Writing – review & editing, Methodology, Formal analysis. **Katarina Patriksson:** Writing – review & editing, Conceptualization. **Heike Rabe:** Writing – review & editing, Conceptualization. **Ola Andersson:** Writing – review & editing, Supervision, Project administration, Funding acquisition, Data curation, Conceptualization. **Katarina Ekelöf:** Writing – review & editing, Writing – original draft, Project administration, Methodology, Formal analysis, Conceptualization.

## Declaration of Competing interest

Maria Wilander reports financial support was provided by Halland Region Health and Care. Ola Andersson reports financial support was provided by The Swedish Society of Medical Research, Southern Healthcare Region. Ola Andersson reports financial support was provided by 10.13039/501100007687Swedish Society of Medicine (Svenska Läkarsällskapet). If there are other authors, they declare that they have no known competing financial interests or personal relationships that could have appeared to influence the work reported in this paper.
