# Supplementary material for: Parental Acceptability and Experiences of In-Bed Resuscitation at Birth: Findings From a Cross-Sectional Survey
Source: J Pediatr Clin Pract. 2026 Jun 12;21:200222. doi: 10.1016/j.jpedcp.2026.200222 (PMC13352167; doi:10.1016/j.jpedcp.2026.200222)
Supplement: Supplementary [file mmc1.docx]

**Supplementary analyses**

The appendix provides complementary analyses and descriptive data supporting the main results presented in the manuscript. Three tables are included to give a more detailed understanding of item-level responses and the robustness of the analytical approach.

Table S1 presents descriptive statistics for individual study items (Likert scale 0-4), reported for the total sample and each group separately: mean (SD), median (Q1–Q3), skewness, kurtosis, and proportions of the lowest/highest responses.

Tables S2 and S3 present the same dichotomized analyses (OR [95% CI]) as the primary analyses but based on alternative group classifications. While the main analyses used parents actually experience (e.g., *in-bed ICR* defined as CC ≥ 180 seconds, or *standard care* with CC within 60 seconds), whereas tables S2 and S3 show corresponding analyses based on parents’ self-reported group affiliation and the allocated group according to the original RCT (intention-to-treat).

Together, these tables provide complementary perspectives on the data and illustrate the consistency of results across different analytic approaches.

| **Table S1. Descriptive statistics for items by group and total sample** | | | | | | | | |
| --- | --- | --- | --- | --- | --- | --- | --- | --- |
|  | Study group | N | Median (Q1-Q3) | Mean (SD) | Skewness | Kurtosis | Strongly positive (%)* | Strongly negative (%)* |
| **Parental acceptance of intervention** |  |  |  |  |  |  |  |  |
| How safe and secure did you feel about your baby receiving support in the warming bed?^1^ | Standard | 114 | 3 (2-3) | 2.6 (1.0) | -0.5 | -0.0 | 18.4 | 3.5 |
| How safe and secure did you feel about your baby receiving support in the birth bed?^1^ | In-bed ICR | 182 | 3 (2-4) | 3.1 (0.9) | -0.5 | -0.8 | 38.7 | 0.0 |
|  |  |  |  |  |  |  |  |  |
| How much visual contact did you have with your baby immediately after birth?^2^ | All | 333 | 3 (1-4) | 2.5 (1.4) | -0.5 | -1.2 | 36.3 | 12.3 |
|  | Standard | 114 | 2 (1-3) | 2.1 (1.4) | -0.1 | -1.3 | 21.9 | 12.3 |
|  | In-bed ICR | 186 | 3 (2-4) | 2.8 (1.5) | -0.8 | 0.8 | 46.2 | 14.0 |
|  |  |  |  |  |  |  |  |  |
| How much were you able to touch your baby immediately after birth?^2^ | All | 333 | 2 (0-4) | 2.0 (1.6) | -0.0 | -1.6 | 29.1 | 28.8 |
|  | Standard | 114 | 1 (0-3) | 1.4 (1.5) | 0.6 | -1.1 | 17.5 | 41.2 |
|  | In-bed ICR | 186 | 3 (1-4) | 2.4 (1.6) | -0.5 | -1.4 | 36.6 | 21.5 |
|  |  |  |  |  |  |  |  |  |
| How did you experience the staff’s communication with you during your baby’s first support minutes?^3^ | All | 333 | 3 (2-4) | 2.9 (1.1) | -0.7 | -0.5 | 37.8 | 3.0 |
|  | Standard | 114 | 3 (2-4) | 2.8 (1.2) | -0.6 | -0.8 | 38.6 | 2.6 |
|  | In-bed ICR | 186 | 3 (2-4) | 2.9 (1.1) | -0.8 | -2.8 | 38.7 | 3.2 |
| **Parental acceptance of research** |  |  |  |  |  |  |  |  |
| How would you describe your feeling about participating in the SAVE study?^4^ | All | 328 | 3 (2-4) | 3.0 (1.2) | -1.2 | 0.6 | 43.5 | 7.5 |
|  | Standard | 113 | 3 (2-4) | 2.6 (1.3) | -0.7 | -0.5 | 29.8 | 12.3 |
|  | In-bed ICR | 182 | 4 (3-4) | 3.3 (1.0) | -1.7 | 2.4 | 53.2 | 4.3 |
|  |  |  |  |  |  |  |  |  |
| What impact do you feel participation in the SAVE study had on your baby’s health?^4^ | All | 333 | 3 (2-4) | 2.8 (0.9) | -0.4 | -0.0 | 25.5 | 1.5 |
|  | Standard | 114 | 3 (2-3) | 2.7 (0.9) | -0.8 | -0.0 | 20.2 | 1.8 |
|  | In-bed ICR | 186 | 3 (3-4) | 3.0 (0.9) | -0.8 | 1.0 | 30.6 | 1.6 |
|  |  |  |  |  |  |  |  |  |
| Given your experience of this study, would you consider participation in another research study in the future?^5^ | All | 333 | 4 (3-4) | 3.3 (0.9) | -1.7 | 3.4 | 52.0 | 3.3 |
|  | Standard | 114 | 3 (3-4) | 3.2 (0.9) | -1.6 | 3.0 | 46.5 | 3.5 |
|  | In-bed ICR | 186 | 4 (3-4) | 3.3 (0.9) | -1.8 | 3.6 | 55.4 | 3.2 |
| Descriptive statistics for study items grouped by “acceptance of intervention” *vs* “acceptance of research” items. Data are shown for total sample and by group according to parents’ actual clinical experience (as-treated). Likert scale 0-4. Responders with uncertain place of resuscitation are not presented.  *Strongly positive and strongly negative indicate the extreme response options for each item.  Response options varied across questions as follows: ^1^Not safe” to Completely safe and secure, ^2^Not at all to To a large extent, ^3^Very poor to Excellent, ^4^Strongly negative to Strongly positive, and ^5^Very unlikely to Very likely. | | | | | | | | |

| **Table S2. Dichotomized comparative analyses by original RCT allocation (intention-to-treat)** | | | | | |
| --- | --- | --- | --- | --- | --- |
|  |  | **Neonatal support in-bed (N=171)  N (%)** | **Neonatal standard support (N=162)  N (%)** | **OR (95% CI)** | ***p*-value** |
| **Parental acceptance of intervention** |  |  |  |  |  |
| How safe and secure did you feel about your baby receiving support in the warming/delivery bed? | “Very safe and secure” to “Completely safe and secure” | 127 (75.1) | 92 (59.0) | 2.10 (1.31-3.38) | 0.002 |
|  | “Not safe and secure” to “moderately safe and secure” | 42 (24.9) | 64 (41.0) |  |  |
|  |  |  |  |  |  |
| How much visual contact did you have with your baby immediately after birth? | “Considerably” to “To a large extent” | 109 (63.7) | 85 (52.5) | 1.59 (1.03-2.47) | 0.04 |
|  | “Not at all” to “To some extent” | 62 (36.3) | 77 (47.5) |  |  |
|  |  |  |  |  |  |
| How much were you able to touch your baby immediately after birth? | “Considerably” to “To a large extent” | 89 (52.0) | 59 (36.4) | 1.89 (1.22-2.94) | 0.004 |
|  | “Not at all” to “To some extent” | 82 (48.0) | 103 (63.6) |  |  |
|  |  |  |  |  |  |
| How did you experience the staff communication with you during the baby’s first support minutes? | “Good” to “Excellent” | 112 (65.5) | 109 (67.3) | 0.92 (0.59-1.46) | 0.73 |
|  | “Very poor” to “Fair” | 59 (34.5) | 53 (32.7) |  |  |
| **Parental acceptance of research*** |  |  |  |  |  |
| How would you describe your feeling about participating in the SAVE study? | “Positive” to “Strongly positive” | 139 (81.3) | 104 (64.2) | 2.66 (1.59-4.44 | < 0.001 |
|  | “Neutral” | 21 (12.2) | 31 (19.1) |  |  |
|  | “Strongly negative” to “Negative” | 11 (6.4) | 27 (16.7) |  |  |
|  |  |  |  |  |  |
| What impact do you feel participating in the SAVE study had on your baby’s health? | “Positive” to “Strongly positive” | 127 (74.2) | 82 (50.6) | 2.81 (1.77-4.46) | < 0.001 |
|  | “Neutral” | 41 (24.0) | 69 (42.6) |  |  |
|  | “Strongly negative” to “Negative” | 3 (1.8) | 11 (6.8) |  |  |
|  |  |  |  |  |  |
| Given your experience of the SAVE study, would you consider participate in another research study in the future? | “Somewhat likely” to “Very likely” | 148 (86.5) | 140 (86.4) | 1.01 (0.54-1.90) | 0.97 |
|  | “Neutral” | 15 (8.8) | 15 (9.3) |  |  |
|  | “Very unlikely” to “Somewhat unlikely” | 8 (4.7) | 7 (4.3) |  |  |
| Table presents odds ratios (95% CI) for dichotomized outcomes (Likert responses 0-2 *vs* 3-4) using the same approach as primary analyses but based on allocated group according to original RCT protocol (ITT).  *Neutral responses within these questions were dichotomized together with negative responses for comparing analyses. | | | | | |

| **Table S3. Dichotomized comparative analyses by parent-reported group affiliation** | | | | | |
| --- | --- | --- | --- | --- | --- |
|  |  | **Neonatal support in-bed (N=153)  N (%)** | **Neonatal standard support (N=180)  N (%)** | **OR (95% CI)** | ***p*-value** |
| **Parental acceptance of intervention** |  |  |  |  |  |
| How safe and secure did you feel about your baby receiving support in the warming/delivery bed? | “Very safe and secure” to “Completely safe and secure” | 117 (78.5) | 102 (58.0) | 2.65 (1.62-4.34) | < 0.001 |
|  | “Not safe and secure” to “moderately safe and secure” | 32 (21.5) | 74 (42.0) |  |  |
|  |  |  |  |  |  |
| How much visual contact did you have with your baby immediately after birth? | “Considerably” to “To a large extent” | 104 (68.0) | 90 (50.0) | 2.12 (1.36-3.32) | < 0.001 |
|  | “Not at all” to “To some extent” | 49 (32.0) | 90 (50.0) |  |  |
|  |  |  |  |  |  |
| How much were you able to touch your baby immediately after birth? | “Considerably” to “To a large extent” | 86 (56.2) | 62 (34.4) | 2.44 (1.57-3.81) | < 0.001 |
|  | “Not at all” to “To some extent” | 67 (43.8) | 118 (65.6) |  |  |
|  |  |  |  |  |  |
| How did you experience the staff communication with you during the baby’s first support minutes? | “Good” to “Excellent” | 105 (68.6) | 116 (64.4) | 1.21 (0.76-1.91) | 0.42 |
|  | “Very poor” to “Fair” | 48 (31.4) | 64 (35.6) |  |  |
| **Parental acceptance of research*** |  |  |  |  |  |
| How would you describe your feeling about participating in the SAVE study? | “Positive” to “Strongly positive” | 127 (83.0) | 116 (64.4) | 2.44 (1.45-4.12) | < 0.001 |
|  | “Neutral” | 15 (0.10) | 37 (20.6) |  |  |
|  | “Strongly negative” to “Negative” | 11 (7.2) | 27 (15.0) |  |  |
|  |  |  |  |  |  |
| What impact do you feel participating in the SAVE study had on your baby’s health? | “Positive” to “Strongly positive” | 120 (78.4) | 89 (49.4) | 3.72 (2.29-6.03) | < 0.001 |
|  | “Neutral” | 29 (19.0) | 81 (0.45) |  |  |
|  | “Strongly negative” to “Negative” | 4 (2.6) | 10 (5.6) |  |  |
|  |  |  |  |  |  |
| Given your experience of the SAVE study, would you consider participate in another research study in the future? | “Somewhat likely” to “Very likely” | 132 (86.3) | 156 (86.7) | 0.92 (0.52-1.82) | 0.92 |
|  | “Neutral” | 13 (8.5) | 17 (9.4) |  |  |
|  | “Very unlikely” to “Somewhat unlikely” | 8 (5.2) | 7 (3.9) |  |  |
| Table presents odds ratios (95% CI) for dichotomized outcomes (Likert responses 0-2 *vs* 3-4) using the same approach as primary analyses but based on parents’ self-reported group affiliation.  *Neutral responses within these questions were dichotomized together with negative responses for comparing analyses. | | | | | |
